# Anatomically Asymmetrical Runners Move More Asymmetrically at the Same Metabolic Cost

**DOI:** 10.1371/journal.pone.0074134

**Published:** 2013-09-24

**Authors:** Elena Seminati, Francesca Nardello, Paola Zamparo, Luca P. Ardigò, Niccolò Faccioli, Alberto E. Minetti

**Affiliations:** 1 Department of Pathophysiology and Transplantation, Faculty of Medicine, University of Milan, Milan, Italy; 2 Department of Neurological and Movement Sciences, School of Exercise and Sport Sciences, University of Verona, Verona, Italy; 3 Department of Pathology and Diagnostics, Section of Radiology, University of Verona, Verona, Italy; University of Utah, United States of America

## Abstract

We hypothesized that, as occurring in cars, body structural asymmetries could generate asymmetry in the kinematics/dynamics of locomotion, ending up in a higher metabolic cost of transport, i.e. more ‘fuel’ needed to travel a given distance. Previous studies found the asymmetries in horses’ body negatively correlated with galloping performance. In this investigation, we analyzed anatomical differences between the left and right lower limbs as a whole by performing 3D cross-correlation of Magnetic Resonance Images of 19 male runners, clustered as Untrained Runners, Occasional Runners and Skilled Runners. Running kinematics of their body centre of mass were obtained from the body segments coordinates measured by a 3D motion capture system at incremental running velocities on a treadmill. A recent mathematical procedure quantified the asymmetry of the body centre of mass trajectory between the left and right steps. During the same sessions, runners’ metabolic consumption was measured and the cost of transport was calculated. No correlations were found between anatomical/kinematic variables and the metabolic cost of transport, regardless of the training experience. However, anatomical symmetry significant correlated to the kinematic symmetry, and the most trained subjects showed the highest level of kinematic symmetry during running. Results suggest that despite the significant effects of anatomical asymmetry on kinematics, either those changes are too small to affect economy or some plastic compensation in the locomotor system mitigates the hypothesized change in energy expenditure of running.

## Introduction

The symmetry between the left and right sides of the body plays an important role in legged locomotion. The symmetrical behaviour of lower limbs during gait has often been taken for granted, mainly for simplicity in data collection and analysis, while the lack of it was frequently considered as an indicator of gait pathology [1]. Differently from what expected, healthy human gait is rather asymmetrical [2], [3]. This seems to reflect a functional difference inherently associated to the laterality of the dominant side characterising each individual [4], [5]. This topic was introduced more than 80 years ago by Lund [6] who showed the effects of structural/anatomical asymmetry on lateral drift in human locomotion. The same experiments were recently repeated and supported the hypothesis of a relationship between leg length inequality and asymmetry in locomotion [7]–[9].

Body symmetry can be further modulated in sports: depending on the discipline, relevant muscles become asymmetrically different (tennis, fencing, throwing, etc.), or they are required to reach similar hypertrophy (ice-skating, downhill skiing, front crawl, etc.) on the two sides of the sagittal plane. Thus, body changes towards or from symmetry are not just the consequence of genetics and laterality, being also caused by specific training protocols.

As the concept of symmetry has an important influence in human locomotion, it plays a key role in the design and maintenance of vehicles, which are periodically inspected and serviced to guarantee wheel balance and homogeneous tyre wearing, in order to reduce fuel consumption and ensure a safe drive. Would it be the same for human running? Can an anatomical/structural asymmetry of the human body cause kinematic/dynamic asymmetry of locomotion? Also, can structural or functional asymmetries be related to some increase of the metabolic cost of transport?

Several authors studied symmetry in locomotion in humans [1]–[4], [10], [11] and also in animals [12], but only few of them investigated the possible interaction between symmetry and energy saving. Manning and collaborators found negative correlations between anatomical symmetry and race time during competitions, both in human running and in galloping horses [13], [14]. These preliminary findings encouraged us to study the possible interactions between different kinds of symmetry (anatomical and dynamical) and the human running performance, not only in term of race time, but also of energy saving. In the present study, we investigate the relationship between the cost of transport (C) while running at different increasing velocities and individual anatomical and dynamical symmetries in three differently trained groups of subjects, with the idea that ‘race cars’ should more strongly rely on symmetry than ordinary ‘automobiles’.

## Materials and Methods

### Subjects

Nineteen healthy male subjects volunteered to participate this investigation. Exclusion criteria included neurological or musculoskeletal pathologies affecting running ability. The institutional ethics committee of the University of Milano had approved all methods and procedures, and subjects gave their written informed consent (approved by the same committee) prior to the start of testing. We clustered participants into three different groups, based on their specific running ability:

- group 1, (n = 7): Untrained Runners (UR), who practiced sport (not specifically running) 3 times *per week* (less than 2 hours *per week*)
- group 2, (n = 7): Occasional Runners (OR), fit athletes, who trained more than 3 times *per week*, (between 2 and 6 hours *per week*). Each of them had previously participated in a national competition (half marathon or 10 km competition)
- group 3, (n = 5): Skilled Runners (SR), master athletes who trained more than 3 times *per week* (at least 6 hours *per week*); they were marathon runners, with a mean performance time of 2 h 44 min 24 s ±10 min 12 s standard deviation (SD).

Anthropometric characteristic of the different subject groups are shown in Table 1.

**Table 1:** Subject characteristics.

	UR	OR	SR
Participants (n)	7	7	5
Age (years)	33.1±13.2	31.9±11.8	42.6±7.4
Body Mass (kg)	70.6±3.4	67.3±6.1	68.2±4.9
Height (cm)	175.9±4.7	177.3±4.0	177.8±4.4
Right leg length (cm)	83.1±3.6	84.0±4.1	85.8±6.3
Left leg length (cm)	82.8±3.7	83.0±3.7	84.8±7.2
Leg length discrepancy (cm)	1.1±0.7	1.0±0.8	1.3±1.0

Number of participants, mean ± SD for age (yrs), body mass (kg), height (cm), right and left leg length (cm), and leg length discrepancy (LLD) (cm) for the 3 different groups of subjects: Untrained runners (UR), Occasional runners (OR) and Skilled runners (SR).

### MR Dataset and 3D Images Processing

In order to evaluate the anatomical symmetries, each participant underwent Magnetic Resonance (MR) imaging. Subjects were adjusted in a supine position as to preserve the maximal body symmetry in the sagittal plane.

MR scans were performed with a 1.5-T superconductive magnet (Siemens, Erlangen, Germany). In all subjects multiplanar T1-weighted Spin-echo sequences were obtained (TE 11, TR 565, flip angle 90°), on a coronal plane for three different anatomical districts: Pelvis district (PD), Upper-Leg district (UD), including thigh and knee, Lower-Leg district (LD), including calf and ankle, with slice thickness of 4 mm. The matrix was 320×320 and the field of view (FOV) was 460×460. Total examination time was less than 7 minutes (36 coronal slices for each district).

**Figure 1:**
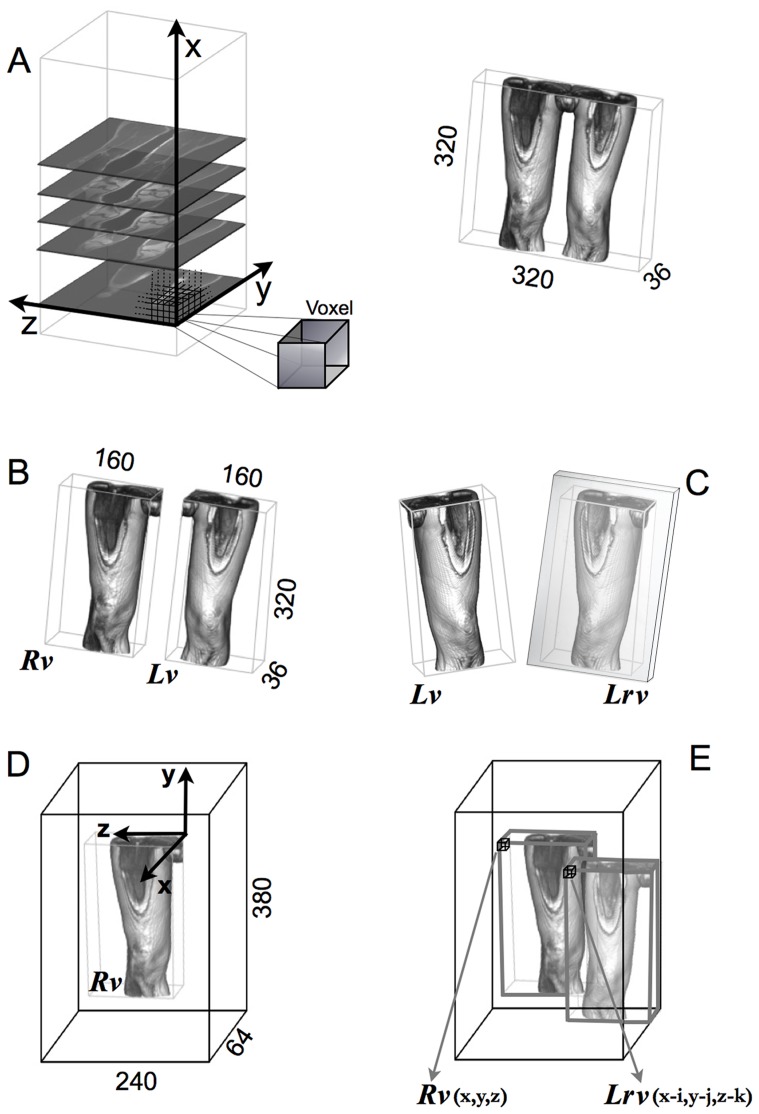
Principal steps involved in the 3D cross-correlation algorithm. a) The 36 slices of the MR sequence, create a 3D volume whose sizes are laterally indicated, b) right volume (*Rv*) and left volumes (*Lv*) separated, c) left reflected volume (*Lrv*) on the sagittal plane (in the mirror), d) zero-padding operation around right volume, e) *Lrv* superimposed to *Rv* in order to find the position that maximize the cross-correlation value.

All the recorded images (saved in DICOM format) were subsequently analyzed with a custom, ad hoc program written in LabVIEW 8.6 (National Instrument, Austin, Texas, USA). The procedure we implemented exports, for each districts, 36 MR images (slices) as two-dimensional matrix of 320×320 pixels, each of which 1.44×1.44 mm, and includes several post-processing steps, as shown in Figure 1.

The 36 coronal slices, for every district, assembled together, re-create a three-dimensional (3D) volume, whose elements (voxel) are values corresponding to a grey level intensity (8 bit scale), reflecting proton density, (Figure 1a). In order to compare the subject’s left lower limb with the right one, firstly, the initial 3D volume has to be split in two separated volumes, right volume (*Rv*) and left volume (*Lv*), (Figure 1b). Successively the *Lv* is specularly reflected, with respect to the sagittal plane (Figure 1c), whilst the *Rv* is bordered by zero intensity voxel (Figure 1d), through a zero-padding operation, so that the left reflected volume (*Lrv*) can be virtually superimposed on the *Rv* (Figure 1e), and moved along the three axes in order to find the best matching overlap and to evaluate the ‘overall’ similarity (i.e. symmetry) between the two limbs. To achieve this aim the algorithm performs a 3D correlation between the contents of the two respective anatomical volumes.

The correlation between two signals (cross-correlation) is a standard approach for signal processing and it has been recently designed in 3D in order to consider simultaneously the full anatomical volume information, to assist radiologists in providing correct diagnosis of metastases within the lungs [15], [16] or brain [17], for instance.

Following Lewis’ approach [18], a normalised cross-correlation coefficient (*r_i,j,k_*), was adopted to identify the symmetry degree between the 3D split volumes:
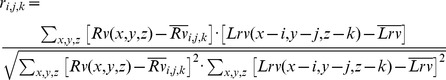
where 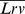 and 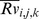 are the voxel mean value of the left reflected volume and the right volume, respectively. The two volumes are virtually superimposed at coordinates i, j and k, and calculations are performed for all pairs of corresponding voxels along x, y and z axes.

For every subject and each anatomical district we evaluated the maximal cross correlation value (*r_max_*) (i.e. the value corresponding to the best overlap between right and left reflected volumes). This coefficient can assume a range of values between −1 and 1, depending upon the similarity of the 3D analyzed volumes, where a value of 1 indicates an exact matching of the *Lrv* with the *Rv*, a value of −1 indicates opposite grey values for voxels in *Lrv* with respect to *Rv*, and a value of 0 indicates no correlation between the two volumes.

**Figure 2:**
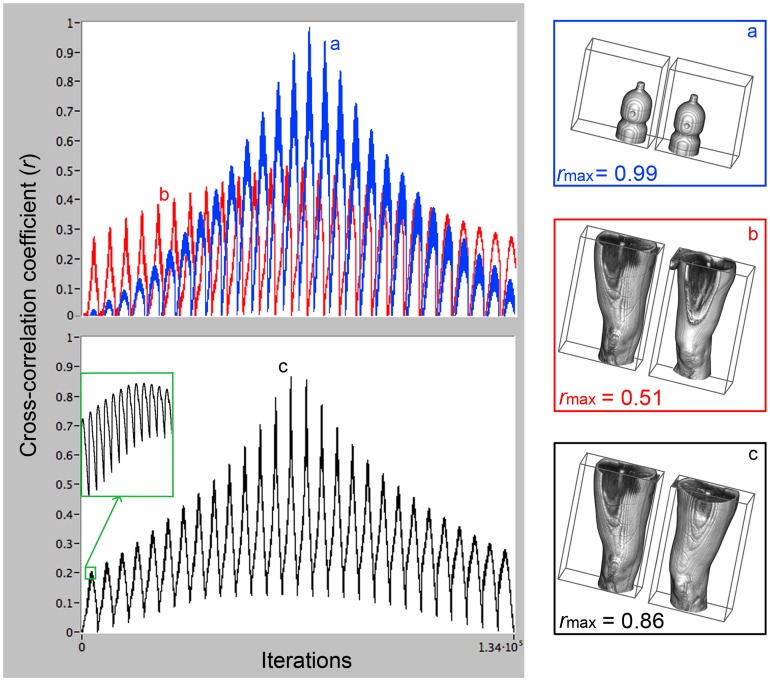
Examples of obtained cross-correlation values plotted versus iterations. Cross correlation values (*r*) of all iterations (134,400 overlap positions = 28 (i)×60 (j)×80 (k), between right volume and left reflected volume); a) comparison between two bottles, filled with the same volume of water (*r*_max = _0.99); b) comparison between the right upper leg of a subject and the left upper leg of a different subject (*r*_max = _0.51); c) comparison between right and left upper legs of the same subject. Inset: enlargement of cross correlation pattern showing the inner processing loop (i coordinates).

Software reliability and accuracy were validated by comparing two identical bottles filled up with water (see Figure 2a), resulting in a maximal cross correlation value of *r*_max_ *= *0.99. The algorithm provided a value of *r*_max_ = 1 only when the right volume of a specific subject was compared with itself, while the lowest value of *r*_max_ was obtained when the right volume of a specific subject was compared with the left reflected volume of an other different subject (*r*_max_ = 0.51, as shown in Figure 2b).

We evaluated for every subject a single maximal cross correlation value (*r*_max_) for each district, (*r*_max_(*PD*) for Pelvis district, *r*_max_(*UD*) for Upper-Leg district and *r*_max_ (*LD*) for Lower-Leg district) and secondly a ‘global’ anatomical cross correlation value (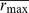) as the mean of the three districts:
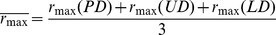



### Kinematics

In order to capture kinematic functional symmetries on many steps, all the subjects performed level shod running on a treadmill (h/p/Cosmos Saturn 4.0, Germany).

Human body has been modelled as a series of linked, rigid segments: 18 reflective markers were placed bilaterally on anatomical landmark points (immediately anterior to ear tragus, shoulder, elbow, wrist, greater trochanter, lateral epicondyle of femur, lateral malleolus, calcaneus, and 5th metatarsal head) and their 3D position was captured at 100 Hz, using an eight-camera Vicon MX optoelectronic system (Vicon, Oxford, UK). In this way, 12 body segments were defined [19].

After a brief period of familiarization on the treadmill, each subject ran at six different incremental speeds: from 2.22 m/s to 5 m/s, step 0.56 m/s. Each speed was maintained for at least 5 min, with a rest period of at least 5 min between successive trials.

The 3D recorded coordinates of the 12 segments, together with the anthropometric tables [20], [21], were used to compute the experimental trajectory of the Body Centre of Mass (BCOM). Successively, we adopted a recent mathematical method [22], [23] simultaneously capturing the spatial and dynamical features of that 3D BCoM trajectory, which allows to quantify dynamical symmetry indices of locomotion in the 3 spatial axes; by having sampled the body motion on a treadmill, the trajectory of the BCOM can be represented by a closed 3D loops (*Lissajous contours*), representing its displacement with respect to the average position. The 3D trajectory is mathematically defined by a 6-harmonic Fourier series, whose coefficients are used to calculate the Dynamical Symmetry Indices 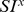 (for progression axis), 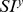 (for vertical axis) and 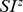 (for lateral axis). The motion of the BCOM is expected to exhibit perfect right–left symmetry if it contained just even harmonics in the progression and y vertical directions, and just odd harmonics in the lateral direction, as within a stride it oscillates twice in the sagittal (y–x) plane and only once in the horizontal (x–z) plane. Dynamical Symmetry indices are then averaged among the strides number (n) as to obtain for each velocity and each subject:
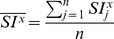


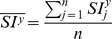


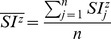



(SI, 0: no symmetry between right and left steps, 1: complete symmetry).

Successively, the three mean dynamic indices (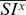, 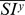, 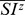) are weighted according to the ‘real’ maximum displacement range of the BCOM, i.e. *dx ( = running speed*X*stride frequency)*, *dy* and *dz*, respectively, and a Global symmetry Index (*GI*) is calculated as
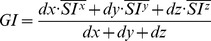



(*GI,* 0: no symmetry between right and left steps, 1: complete symmetry).

### Energy Cost Measurement

Oxygen consumption (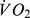) of running was measured with a breath-by-breath gas analyzer (Cosmed K4b^2^, Rome, Italy). Data, including heart rate (HR), were recorded at each progression speed, after the metabolic steady state had been achieved (3 min), for further 2 minutes. 5 minutes of testing was performed at each speed. Resting 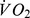 was measured while standing. Respiratory Exchange Ratio (RER) was monitored in order to check for aerobic conditions (RER<1). We expressed the metabolic Cost of Transport (C), i.e. the oxygen consumed to move 1 kg of body mass 1 m distance, in J (kg m)^−1^ by dividing the net 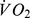 [measured - resting, [ml O_2_ (Kg min)^−1^] by the progression speed (m min^−1^), and by assuming an energy equivalent of 20.9 J ml O_2_^−1^.

### Statistical Analysis

Relationships between variable pairs were investigated using Pearson’s correlation coefficient. To compare speed dependent variables (C, HR, 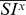, 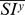, 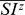 and *GI*), differences were analyzed using a two-ways ANOVA (groupxspeed) (with a post-*hoc* Bonferroni correction). For speed independent variables (*r*_max_(*PD*), *r*_max_(*UD*) and *r*_max_(*LD*)), we performed a one-way ANOVA for repeated measures in order to detect difference among districts. Furthermore, Principal Component Analysis (PCA) was performed on the three anatomical indices, in order to estimate their relative contribution to the total variance.

Statistical significance was accepted when p<0.05.

## Results

Since only five OR and five SR subjects were able to complete all the running protocols up to 5.0 m/s, and UR subjects stopped at the speed of 4.44 m/s, we did not consider in the statistical analysis the highest speed level.

### Anatomical Symmetries

Anatomical symmetries are described by the maximal cross-correlation value for each district (*r*_max_(*PD*), *r*_max_(*UD*) and *r*_max_(*LD*)), and by the global anatomical cross correlation value (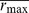). These values are limited to only 17 subjects, because two MR tests (one for the UR and one for the SR) had to be discarded due to technical problems.

One-way ANOVA between the three groups of subject didn’t show any difference between UR, OR and SR for the cross-correlation values, while we found significantly lower values of anatomical symmetry for pelvis district, compared to the upper (p<0.05) and lower leg district (p<0.01) (*r*_max_(*PD*) = 0.77±0.09, *r*_max_(*UD*) = 0.82±0.05 and *r*_max_(*LD*) = 0.83±0.05). PCA showed that 65.8% of the total variance was explained by the first principal component, where the three considered parameters (*r*_max_(*LD*), *r*_max_(*UD*) and *r*_max_(*PD*)) had almost the same weight. However UD seems to give the greatest contribution to the first principal component, with respect to the other two districts.

**Figure 3:**
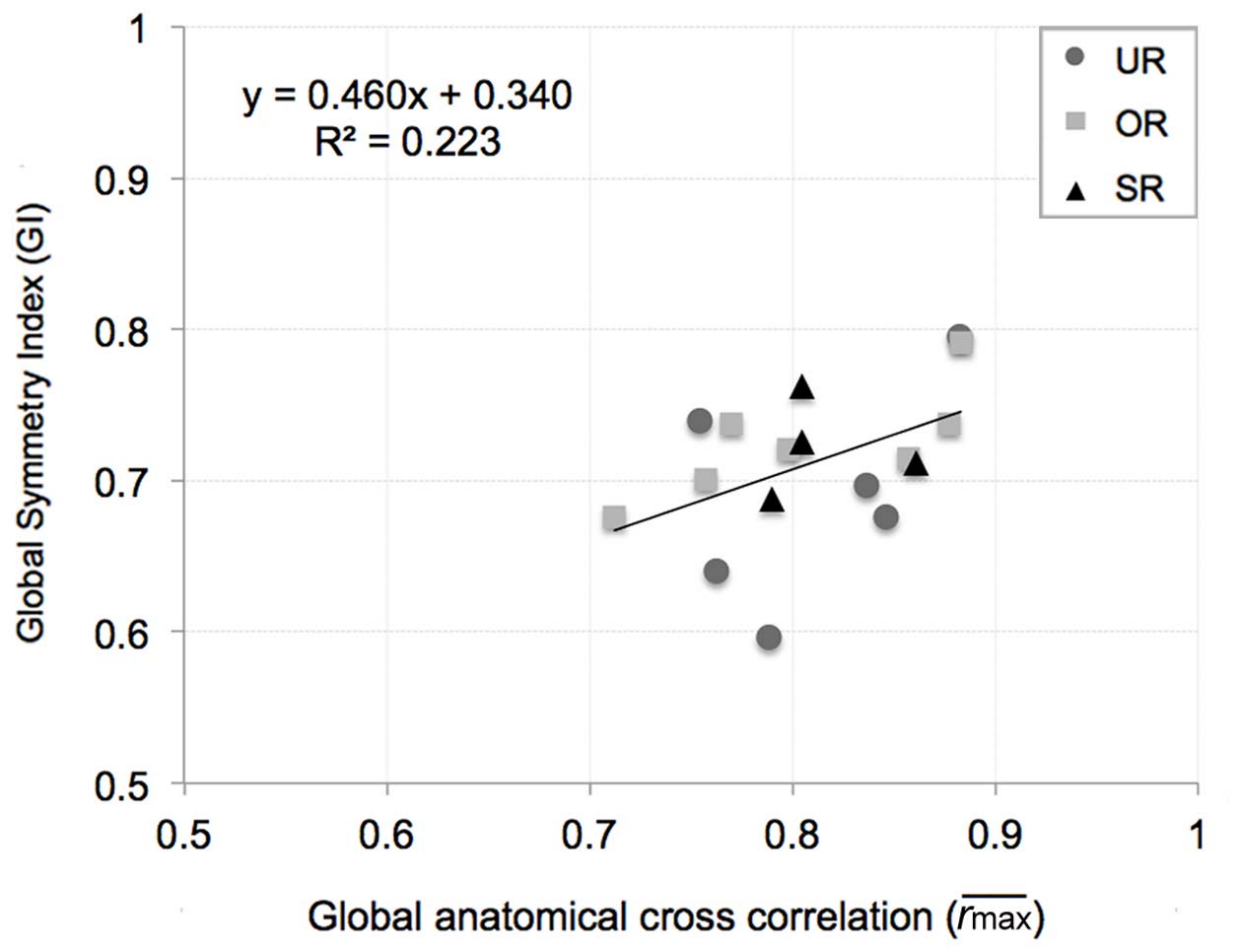
Regression of the mean dynamic Global Symmetry Index (*GI*) versus the global anatomical cross correlation value (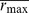). Each point represents the mean Global Symmetry Index averaged among the different running speeds for each subject; Untrained Runners (UR), Occasional Runners (OR) and Skilled Runners (SR) (r = 0.473; p = 0.055).

Results regarding pairwise correlations between variables are summarized in Table 2: *r*_max_(*UD*) is significantly correlated with *r*_max_(*PD*) and *r*_max_(*LD*) (p<0.05), also *r*_max_(*PD*) and *r*_max_(*LD*) seem to be positively correlated even if not significantly (p = 0.087). Significant results were found also between anatomical symmetries and kinematics (mean values for the Global Symmetry Index (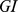) were evaluated starting from the single values of 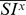, 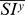 and 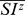 and averaged within each group of speeds for each subject); in particular *r*_max_(*PD*) is positively correlated with 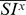 (p<0.01) and 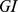 (p<0.05) and also 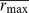 is positively and significantly correlated with 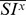 (p<0.05)_,_ while we observed a positive trend between 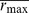 and *GI*, even if not significantly (p = 0.055) (see Figure 3).

**Table 2:** Statistical correlation matrix results between variable pairs.

		Anatomical Symmetry				Dynamical Symmetry				_Economy_
		*r*_max_ *(PD)*	*r*_max_ *(UD)*	*r*_max_ *(LD)*	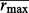	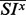	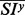	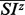	*GI*	C
**Anatomical** **Sym.**	***r***_max_ ***(PD)***	1	0.501*; **0.040**	0.427;0.087	0.871**; 0.000	0.651**; 0.005	0.110;0.675	−0.094;0.719	0.606**; **0.010**	0.157;0.547
	***r***_max_ ***(UD)***		1	0.507*; **0.038**	0.782**; **0.000**	0.322;0.208	0.003;0.990	0.020;0.940	0.357;0.160	−0.114;0.662
	***r***_max_ ***(LD)***			1	0.748**; **0.001**	0.045;0.863	0.103;0.694	0.304;0.236	0.046;0.860	0.059;0.822
	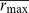				1	0.487*; **0.048**	0.095;0.716	0.055;0.834	0.473;0.055	0.072;0.785
**Dynamical** **Sym.**	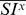					1	0.186;0.447	0.009;0.972	0.992**; **0.000**	0.005;0.983
	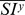						1	0.617**; **0.005**	0.186;0.445	0.105;0.668
	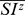							1	0.012;0.959	0.211;0.385
	***GI***								1	−0.001;0.995
**Economy**	**C**									1

Pearson Correlation coefficient is presented together with the relative p-value for the following parameters: maximal cross correlation values for each anatomical district (*r*_max_(*PD*), *r*_max_(*UD*) and *r*_max_(*LD*)), global anatomical cross correlation value (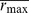), dynamical symmetry indices for each direction (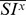
_,_ 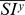 and 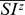
_)_, Global Symmetry Index (*GI*) averaged among the different running speeds for each subject and metabolic Cost of transport (C). Values in bold indicate significant correlations (* = p<0.05, ** = p<0.01).

### Kinematics

**Figure 4:**
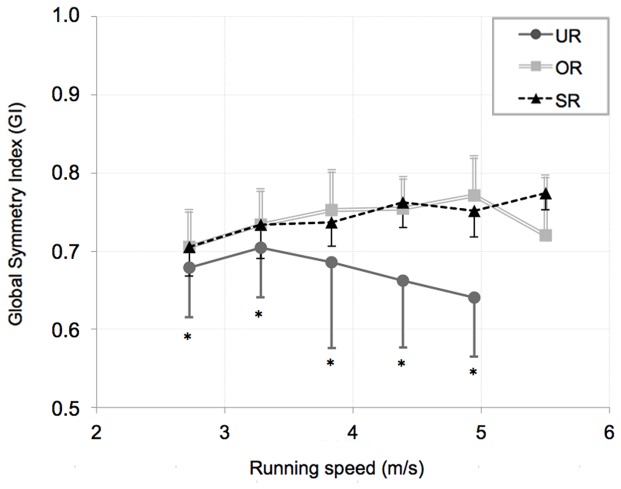
Mean values for the dynamic Global Symmetry Index (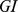) plotted against running speed. Mean values for the dynamic Global Symmetry Index (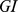) are evaluated starting from the single values of 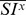, 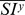 and 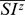 and averaged within each group of subjects, ± SD, in untrained runners (UR), occasional runners (OR) and skilled runners (SR). Two-way ANOVA (group×running speed) show that the group of UR had a mean *GI* always lower compared to the OR and SR, (* = p<0.01), independently from the running speed.

Mean values for the Global Symmetry Index (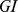), evaluated starting from the single values of 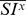, 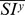 and 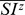 and averaged within each group of subjects, ± SD, are shown in Figure 4. We performed a two-ways ANOVA, where independent variables were running speed and subject group and the dependent variable was *GI*. Results show that UR have a *GI* significantly lower than both OR and SR at each velocity (p<0.01). Also, *GI* for UR seems to decrease with increasing running velocity, even if not significantly. Statistical analysis did not show any difference between UR, OR and SR for the single kinematic symmetry indices, while one-way ANOVA for repeated measure shown significantly lower values for 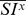, (0.72±0.06) compared to 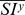 (0.88±0.04) and 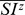 (0.85±0.05), (p<0.01).

### Cost of Transport

**Figure 5:**
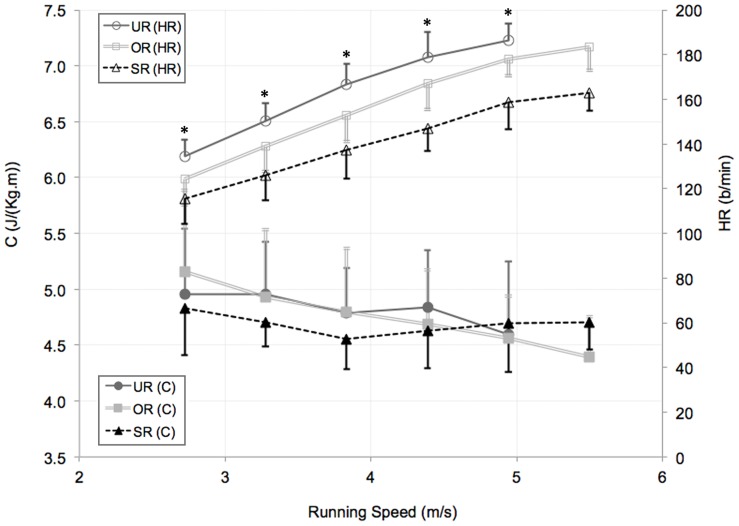
Mean values ± SD for the cost of transport (C) (lower curves) and for the heart rate (HR) (upper curves). C and HR are plotted against running speed for untrained runners (UR), occasional runners (OR) and skilled runners (SR). Results obtained with the two-way ANOVA (group×running speed) show no significant difference among groups of subjects across velocity for C, which results to be independent of the running speed. HR increased significantly with the running speed for all the three group of subjects. Furthermore we obtained significantly higher HR values for UR compared to OR and SR (* = p<0.01).

Results for the metabolic cost C and HR are presented in Figure 5. C is confirmed to be independent of speed, with no differences among running groups. At the same speed, HR decreased as runners’ ability increased, with values for SR significantly lower than for OR and UR. No significant correlation was found between the C and the previously analysed parameters, both for kinematics and for anatomical values (Table 2).

## Discussion

The main aim of this project was to investigate the relationship among the anatomical/structural symmetry of the lower limbs, the dynamical symmetry of the 3D BCOM displacement and the metabolic cost of human running. C has been considered as an indirect index of running performance: at the same sustainable fraction of maximal 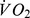, the lower the cost the higher the average speed [24]. While being aware of the speed and training level independency of C, as debated and reported in the literature [25]–[29], our hypothesis was that more asymmetrical limbs, in subjects committed to run with symmetrical steps, would have involved a higher C. In other words, part of the inter-subject C variance could have been explained by different level of anatomical asymmetry.

Differently from previous studies dealing with gross morphological features (bones length [5], [8], human face [13] and horse muzzle [14] landmarks) and isolated gait parameters (stride length and frequency [30], [31], joint angles [3] and ground reaction forces [10]), we analysed the symmetry of the ‘whole’ (left and right) lower limb anatomy and of the global running kinematics (3D trajectory of BCOM), in three groups of differently trained athletes. Our hypothesis, inspired by the engineering of motor vehicles, was not completely verified. C was not significantly correlated either with anatomical symmetries or with dynamical symmetries in running, while we found significant correlations between the anatomical and dynamical symmetries indices (Table 2). This indicates that the more anatomically symmetrical are the subjects, the more symmetrical is their running gait (especially in the forward (x) direction).

This finding is in accordance with the recent literature, where high level of leg length discrepancy (LLD) is correlated with low symmetrical gait coefficients [7] in walking. In our work, individual LLD was always lower than 2 cm (Table 1), and had no effect on C, according to the studies of Gurney [32].

It is possible that some physiological adaptations of the human machinery compensate for small asymmetries typical of the mechanics of our legged system [1], [2], with no influence on C. Rather, larger anatomical discrepancies, like a LLD higher than 2 cm [32] or a body mass not uniformly distributed [33], [11], could influence economy. Similar adaptations behaviours might have occurred in runners wearing new and worn shoes [34], or on surfaces of different stiffness [35]. Despite of the changed properties of materials, runners modified their motion pattern as to retain their original dynamics of running.

This could occur also in the subjects of this study, who seem to compensate their anatomical body asymmetries and minimize C, a strategy frequently adopted by animals [36]. With the main propulsive muscles operating close to isometric in running [37], tendons can store (stretching) and release (shortening) variable amounts of elastic energy during each step, in the attempt to adapt to different anatomical asymmetries. In this way the metabolic cost can be potentially kept unchanged.

In addition, although HR results (Figure 5) witness the appropriateness of clustering subjects according to the different training status (most skilled runners reported the lowest HR, at the same speed, p<0.01), the almost speed-independent C values seem not to be influenced by the different fitness level, as also found by other investigators [27], [29].

As also indicated in previous studies, training and experience seem to be important elements in the lower limb joint angle symmetry and in the stride variability of running, even at no apparent metabolic benefit [29]–[31]. The most experienced and high performance athletes can maintain, even at high velocities, higher dynamical symmetry than untrained runners (Figure 4). As step frequency and muscles effort increase, the higher physical demand and peripheral fatigue could impair the maintenance of a symmetrical gait and a consistent locomotion pattern, as seen for the UR group.

Furthermore, MRI measurements showed that the anatomical symmetry does not depend on the investigated district. PCA and correlation among lower limb districts could have been caused by misalignments of the two limbs during MRI test. However, due to the use of alignment tools during the tests, we feel confident that the intra-subject symmetry correlation among districts is not a measurement artefact. Similar eigenvalues from PCA suggest that the total variance of symmetry is equally explained by the three districts.

This work brings developments in the study of locomotion symmetry, also by means of newly introduced methodologies (BCOM 3D trajectory analysis and 3D cross-correlation between ‘whole’ limb MRI voxels). Differently from the original hypothesis, asymmetrical limbs generate asymmetrical body running at no apparent additional metabolic cost. This suggests some plasticity of the human body in coping with structural changes, with the final result of preserving locomotion economy. Deeper insights have been obtained regarding the relationship between the symmetries correlation residuals and the cost of transport, with the idea that subjects would be less economic when their anatomical and dynamical symmetry values do not match. Supplemental analysis and discussion regarding this hypothesis have been reported in the Appendix S1. Even if statistical results in this perspective are weak, possibly due to the relatively small sample size and low asymmetry level, there are some hints suggesting that only the runners who fail to match their anatomy and dynamics features have an increased cost of locomotion. Therefore, the initial hypothesis embedded in the title “anatomically asymmetrical runners move more asymmetrically at the same metabolic cost” is still valid (i.e. the cost would increase when an anatomically asymmetrical runner attempts to move in a symmetrical way). Further studies focusing on adaptations of the muscle-tendon interplay could reveal how human machine compensate the small structural asymmetries that characterize our legged system. The anatomical asymmetry threshold, above which the now expected asymmetrical gait will also involve an increase in running cost, is the challenge for future investigations.

## Acknowledgments

The authors would like to thank all the subjects for their participation in the study, and the Technician Lauro Dalla Chiara for his help during the MR scans performed at the University Hospital Polyclinic “Borgo Roma” in Verona (Italy). Statistical support from Carlo M. Biancardi is also greatly appreciated.

## Supporting Information

Figure S1
**Examples of univariate and bivariate regressions.** Four different types of linear regressions are presented as examples of correlation between Dynamical Symmetry index in forward direction (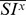) and maximal cross-correlation value for Pelvis District (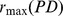): a) Univariate regression, b) Univariate regression with intercept forced to be equal to 0, c) Bivariate regression, d) Bivariate regression with intercept forced to be equal to 0. Untrained Runners (UR), Occasional Runners (OR) and Skilled Runners (SR) symbols as in Figure 3. N.B. The determination coefficient in regressions lines forced through the origin, differently from the general model, does not reflect the fraction of the variability in the dependent variable explained by the independent variable. This makes R^2^ values unrealistically high and not comparable with the ones obtained in the general models.(TIF)

Appendix S1 (DOCX)
