# Supplementary figures and images for: Anatomically Asymmetrical Runners Move More Asymmetrically at the Same Metabolic Cost
Source: PLoS One. 2013 Sep 24;8(9):e74134. doi: 10.1371/journal.pone.0074134 (PMC3782489; doi:10.1371/journal.pone.0074134)

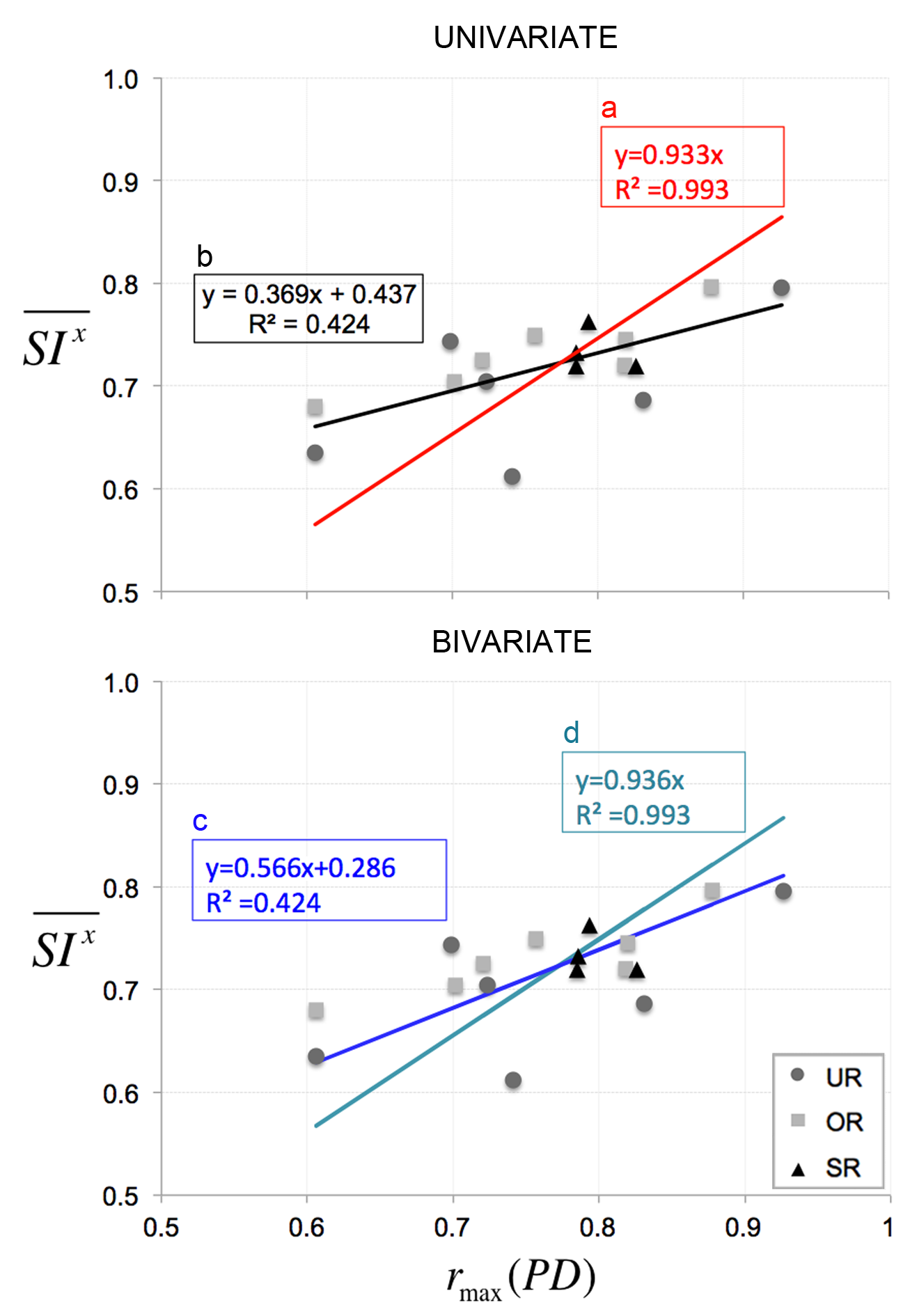

Supplement: Figure S1 — Examples of univariate and bivariate regressions. Four different types of linear regressions are presented as examples of correlation between Dynamical Symmetry index in forward direction () and maximal cross-correlation value for Pelvis District (): a) Univariate regression, b) Univariate regression with intercept forced to be equal to 0, c) Bivariate regression, d) Bivariate regression with intercept forced to be equal to 0. Untrained Runners (UR), Occasional Runners (OR) and Skilled Runners (SR) symbols as in Figure 3. N.B. The determination coefficient in regressions lines forced through the origin, differently from the general model, does not reflect the fraction of the variability in the dependent variable explained by the independent variable. This makes R2 values unrealistically high and not comparable with the ones obtained in the general models. (TIF) [file pone.0074134.s001.tif]
