# Supplementary material for: Anatomically Asymmetrical Runners Move More Asymmetrically at the Same Metabolic Cost
Source: PLoS One. 2013 Sep 24;8(9):e74134. doi: 10.1371/journal.pone.0074134 (PMC3782489; doi:10.1371/journal.pone.0074134)
Supplement: Appendix S1 — (DOCX) [file pone.0074134.s002.docx]

**Appendix S1**

Pairwise correlation between variables regarding anatomical symmetry, dynamical symmetry (kinematics) and running economy (C), are reported in Table 2 of the paper. While results indicate a positive trend between anatomical maximal cross correlation values and dynamical symmetry indices, no significant correlation was found between symmetry and the cost of transport C. It is possible though, that the correlation between asymmetry and metabolic cost doesn’t completely explain our finding. For these reasons we calculated the “residuals” of the dynamic vs. anatomic similarity correlation. These “residuals” represent how far is a subject from the best match between dynamical and anatomical symmetries (or asymmetries). Thus, we investigated the possible relationship between those “residuals” and the cost of transport C.

We considered four pairs of variables (dynamical vs. anatomical), positively correlated as reported in Table 2 in the manuscript. By considering dynamical indices as the dependent variables and anatomical indices as the independent variables, we summarised these results in Table S1.

Although p-value and Pearson correlation coefficient (R) indicate a positive relationship between dynamical and anatomical indices for each of these pairs, analysis of “residuals” depends on the types of linear regression that can be adopted.

1. *Univariate regression*: the dependent variable is the dynamical variable ($\overline{SI^{x}}$or GI). i.e. it could be predicted by the anatomical variable ($\overline{r_{\max}}$or $r_{\max}(PD)$). In this case “residuals” are evaluated as the vertical distance from each single point to the regression line.
2. *Univariate regression with intercept equal to 0:* Also in this case the dynamical variable is predicted by the anatomical variable and the “residuals” are evaluated in terms of vertical distance, but the regression line is forced to pass thought the origin, because we suppose that totally dynamically asymmetrical subjects should be associated to complete anatomical asymmetry.
3. *Bivariate regression:* neither the dynamical variable depends of the anatomical variables, nor the anatomical variable depends of the dynamical variable. The “residuals” in this case are evaluated as the orthogonal distance from each point to the regression line.
4. *Bivariate regression with intercept equal to 0:* As in the previous case there is no 'cause-effect' dependence between variables. The “residuals” are evaluated in term of orthogonal distance, but the regression line is forced to pass thought the origin, because we suppose that totally dynamically asymmetrical subjects should be associated to complete anatomical asymmetry.

We provided an example of the four regression types for the variables $\overline{SI^{x}}$ and $r_{\max}(PD)$ in Figure S1. As you can see in the graphs of this figure, we obtained 4 different lines depending on the regression type we chose. We performed these test for all the variable pairs listed in Table S1, resulting in 16 groups of “residuals”. After that we performed a univariate regression between the Cost of transport (dependent variable) and the “residuals” (independent variable). Data analysis has been performed with SMATR software (Version 2, Oct. 2006), implemented by Falster D., Wright I. (University of New South Wales) and Warton D. (Macquarie University).

Although in most cases we found C to be positively correlated with the “residuals”, significant values were obtained only when performing *univariate regression* (i.e. considering dynamical symmetry depending on anatomical symmetry) of *GI* vs. $r_{\max}(PD)$.

The whole statistical correlation matrix for *univariate regression* (between “residuals” and COST) is reported in Table S2.

This positive trend between “residuals” and Cost of transport could suggest, that only runners who fail to match their anatomy to gait dynamical features have an increased cost of locomotion. Despite this weak statistical support, we feel the paper title we propose still reflects the actual findings.

It is interesting to notice that the only significant correlation regards the univariate regression between the dependent variable C and the “residuals” between the Global Symmetry Index (*GI*) and the maximal cross-correlation values for the Pelvis District ($r_{\max}(PD)$). This anatomical zone is the most asymmetrical compared to the others, supporting the reasons for a significant correlation.

We didn’t choose the subjects of the three groups according to a set range of asymmetries. They were individuals of different fitness level, with respect to running performance, with an expected low range of anatomical asymmetry. Therefore the effects on cost were supposed to be small because of the inclusion criteria. It is possible that a higher sample size and a different choice of subjects would increase the regression power and substantiate other functional/structural relationships.

**Table S1. Pairs of anatomical/dynamical variables:**

|  | **Dynamical index** | **Anatomical index** | **R** | **R^2^** | **p-value** |
| --- | --- | --- | --- | --- | --- |
| Pair 1 | $\overline{SI^{x}}$ | $r_{\max}(PD)$ | 0.651 | 0.424 | 0.005** |
| Pair 2 | $\overline{SI^{x}}$ | $\overline{r_{\max}}$ | 0.487 | 0.235 | 0.048* |
| Pair 3 | *GI* | $r_{\max}(PD)$ | 0.606 | 0.368 | 0.010** |
| Pair 4 | *GI* | $\overline{r_{\max}}$ | 0.473 | 0.224 | 0.055 |

The 4 pairs of variables (dynamical indices vs. anatomical indices) positively correlated are listed together with Pearson correlation coefficient (R), coefficient of determination (R^2^) and statistical significance (p-value), (* = p < 0.05, ** = p < 0.01).

**Table S2. Statistical correlation matrix results between “residuals” and Cost of Transport:**

|  | **COST (C)** | |
| --- | --- | --- |
|  | **R** | **p-value** |
| **Residuals$\overline{SI^{x}}$vs.** $r_{\max}(PD)$ | 0.481 | 0.05 |
| **Residuals *GI* vs.** $r_{\max}(PD)$ | **0.498** | **0.042*** |
| **Residuals *GI* vs.** $\overline{r_{\max}}$ | 0.427 | 0.088 |
| **Residuals$\overline{SI^{x}}$vs.** $\overline{r_{\max}}$ | 0.408 | 0.104 |

Pearson correlation coefficient (R) is presented together with the relative p-value the four univariate regressions (* = p < 0.05).
